# Adaptive outdoor physical activities for adults with mobility disability: a scoping review

**DOI:** 10.3389/fresc.2023.1331971

**Published:** 2024-01-08

**Authors:** Pegah Derakhshan, William C. Miller, Andrea Bundon, Delphine Labbé, Tanelle Bolt, W. Ben Mortenson

**Affiliations:** ^1^ GF Strong Rehabilitation Centre, University of British Columbia, Vancouver, BC, Canada; ^2^ Graduate Program in Rehabilitation Sciences, Faculty of Medicine, University of British Columbia, Vancouver, BC, Canada; ^3^ International Collaboration on Repair Discoveries (ICORD), Vancouver, BC, Canada; ^4^ Department of Occupational Science and Occupational Therapy, Faculty of Medicine, University of British Columbia, Vancouver, BC, Canada; ^5^ School of Kinesiology, University of British Columbia, Vancouver, BC, Canada; ^6^ Department of Disability and Human Development, University of Illinois at Chicago, Chicago, IL, United States; ^7^ Founder of RAD, Recreational Adaptive Society, Invermere, BC, Canada

**Keywords:** outdoor physical activity, adaptive devices, mobility impairment, scoping review, Universal Design (UD), barriers and facilitating factors, participation and inclusion

## Abstract

**Introduction:**

Outdoor physical activity (PA) contributes to the physical and mental health and well-being of individuals with a mobility impairment. However, individuals are commonly excluded from outdoor PA because of accessibility challenges. No reviews summarizing evidence on factors that facilitate/hinder participation and inclusion of individuals with mobility disabilities in adaptive outdoor PA were identified.. This makes it challenging to establish the key components for implementing inclusive outdoor PA interventions. A scoping review was conducted to identify barriers and facilitators to participation in adaptive outdoor PA and identify suggestions for adaptive outdoor PA design.

**Methods:**

A scoping review of qualitative and quantitative studies was conducted based on the methodological framework of Arksey and O'Malley with modifications by Levac. Barriers and facilitators were categorized into four levels based on a Social Ecological Model (SEM). Suggestions for interventions designed to overcome accessibility issues of outdoor PA were classified based on Universal Design (UD).

**Results:**

Thirty-seven factors regarding barriers and facilitators of outdoor adaptive PA were extracted from 19 studies published between 2002 and 2023. Barriers and facilitators were identified primarily in four levels of the SEM, including intrapersonal, social-environmental, physical-environmental, and policy-related. Eleven design suggestions were identified and categorized according to the seven principles of UD. This study identified gaps in the presented barriers and facilitators and the design suggestions of the included studies, mainly at the social and environmental level, such as a lack of innovation in program delivery and logistics.

**Conclusion:**

This study identified gaps in knowledge about facilitators and barriers to outdoor adaptive PA and in the design of interventions addressing them. Future research should focus on the strategies addressing these gaps by involving individuals with mobility disability in designing interventions to gain a better insight into their needs.

## 1 Introduction

Outdoor physical activities (PA) contribute to the physical, mental, and emotional well-being of all individuals (1–3), including individuals with a mobility disability who constitute 10% and 12.1% of adults in Canada and the United States, respectively (4, 5). In addition, previous studies indicate that individuals with disabilities show higher levels of accomplishment and growth while taking part in outdoor activities compared to individuals without disabilities (6). Outdoor PA provides benefits comparable to indoor PA but with additional advantages such as enhanced mood and heightened relaxation (7–9). Outdoor PA can also protect against cardiovascular disease (10). Moreover, individuals are often more motivated to take part in outdoor physical activities than indoor activities because of the inherent appeal of a natural environment (11).

Taking part in PA is a fundamental human right, enshrined in the United Nations Convention on the Rights of Persons with Disabilities (UNCRPD) (12). Six universal principles were introduced by the UNCRPD, including accessibility, autonomy, non-discrimination, equality of opportunities, inclusion, and independence (12). These principles, agreed to by 184 nations, including Canada, entail the obligation to improve existing legislature, integrating them into practice, as well as applying emerging technologies and new design interventions to facilitate the well-being and quality of life of individuals with mobility disabilities to ensure access to outdoor activities.

Despite this emphasis by the United Nations, individuals with disabilities are commonly excluded from outdoor physical activities, primarily because of accessibility challenges (6, 13). Multiple studies identified lists of different facilitators and barriers to participation, such as a lack of awareness of the existence of outdoor recreation programs, limited access to the necessary equipment, and insufficient environmental accessibility (14, 15). Although the information on facilitators and barriers to participation is enlightening in itself, organizations need to apply and use this information as a basis for selecting, designing, and implementing strategies to increase participation in adapted outdoor PA (16).

To promote non-discriminatory design of programs and interventions for adaptive outdoor PA, a variety of different approaches are used, including ability-based design (17), inclusive design (18), barrier-free design (19), and design for all (20). Although these are different concepts, all have the same overarching goal: to provide the most usable and effective opportunities for all who could use the system, regardless of any challenges they may face (21). At this time, no consensus exists on how to formulate the concept of accessibility in diverse areas. However, the concept of Universal Design (UD) (22), which is advocated for in the UNCRPD (12) as a means of addressing accessibility issues in the design of new programs, can be considered as guidance for addressing challenges when designing inclusive outdoor physical activities. As the UNCRPD is part of a larger paradigm shift from understanding disability as “located” in the body of a person towards an understanding of disability as the result of individuals with impairments and their encounters with attitudinal and environmental barriers, UD was proposed as an approach with the potential to empower individuals, dismantle barriers, and create suitable environmental conditions for the social inclusion of everybody, regardless of their abilities (23–25). UD has seven categories or principles that guide the design process, including (1) equitable, (2) flexible, (3) simple and intuitive, (4) perceptible information, (5) tolerance for error, (6) low physical effort, and (7) size and space for approach and use. These principles guide the design process to ensure that the design is useful, adaptable, understandable, effective, safe, comfortable, and spacious for diverse users.

Even though various programs have applied different concepts to design inclusive and adaptive outdoor physical activity interventions, to our knowledge, a systematic analysis of studies and interventions with the goal of synthesizing evidence on adaptive outdoor PA design that facilitates/hinders participation and inclusion has not yet been conducted. The lack of evidence to inform best practices in inclusive and adaptive outdoor physical activity design makes it challenging for program developers to create inclusive outdoor physical activity interventions and programs (16, 26).

For this reason, the present study aimed to review empirical literature about the design of inclusive outdoor physical activity for people with disabilities. The study had three main objectives: To (a) identify facilitators and barriers to participation in adaptive outdoor physical activity, (b) identify design recommendations for adaptive outdoor physical activities, and (c) categorize these recommendations based on the seven UD principles.

### 1.1 Theoretical framework

Physical activity occurs as a result of a complex interplay of personal and environmental factors (27). The *Social Ecological Model* (SEM) (28) describes the multifaceted network of parameters that affect choices of behavior through interactions between the environment and an individual (29). This model displays both the settings in which the individual participates and those that affect them, even if they don't directly participate (29). Depending on what the particular problem or situation requires, the conceptualization of the SEM can vary between three and five layers (29). A four-layer version of the model proposed by McLeroy and colleagues in 1988 includes the layers of intrapersonal, interpersonal, organizational, and community factors. Central to this model is the individual with their attitude, knowledge, and skills. The other levels are social and physical levels, and they include *interpersonal factors* covering the relationships and interactions that an individual has with other people, such as their family, friends, peers, and co-workers; *organizational factors*, referring to the institutions and organizations that an individual belongs to or interacts with, such as schools, workplaces, or health care facilities, and *community factors*, encompassing the broader social and physical context that an individual lives in, such as their neighborhood, city, or country.

The different layers of the SEM are interconnected and have a dynamic relationship with each other, collectively influencing individual and collective behaviors and health outcomes (30). Different levels of SEM models interact in the creation of environments and programs that promote physical activity and health behaviors in general. Studying those levels can help to form an understanding of the determinants of participation, as shown by several studies. These studies suggested using customized SEMs to examine participation in sport and recreation across a variety of domains and activities to gain a clearer understanding of participation (29, 31). Bauman et al. (31) used an SEM for outdoor activity. In addition to interpersonal and social factors, their model further elaborates on the role and importance of the natural and built environment, as well as the equipment in the physical environment and policies (31). Furthermore, by removing barriers to involvement, the model may aid the development of programs and policies that strengthen participation (16). This allows the SEM model to combine with other models, such as Social Cognitive Theory (SCT) (32) and the Health Action Process Approach (HAPA) (33) that can be useful for developing adaptive outdoor physical activity interventions that are implemented at one specific level (e.g., policy) to address factors at the intrapersonal as well as interpersonal levels (16). In this context, it will be helpful to divide the social level into two subcategories, namely intrapersonal and institutional/community factors (34). Thus, in this study particular case, the application of such a model to the study of adaptive outdoor activities, a SEM was used with four levels, starting with intrapersonal factors, then social environmental (interpersonal and institutional), physical environmental factors (built environment, natural environment and equipment), and finally policy/regulatory factors (Figure 3A).

## 2 Methods

**Figure 1:**
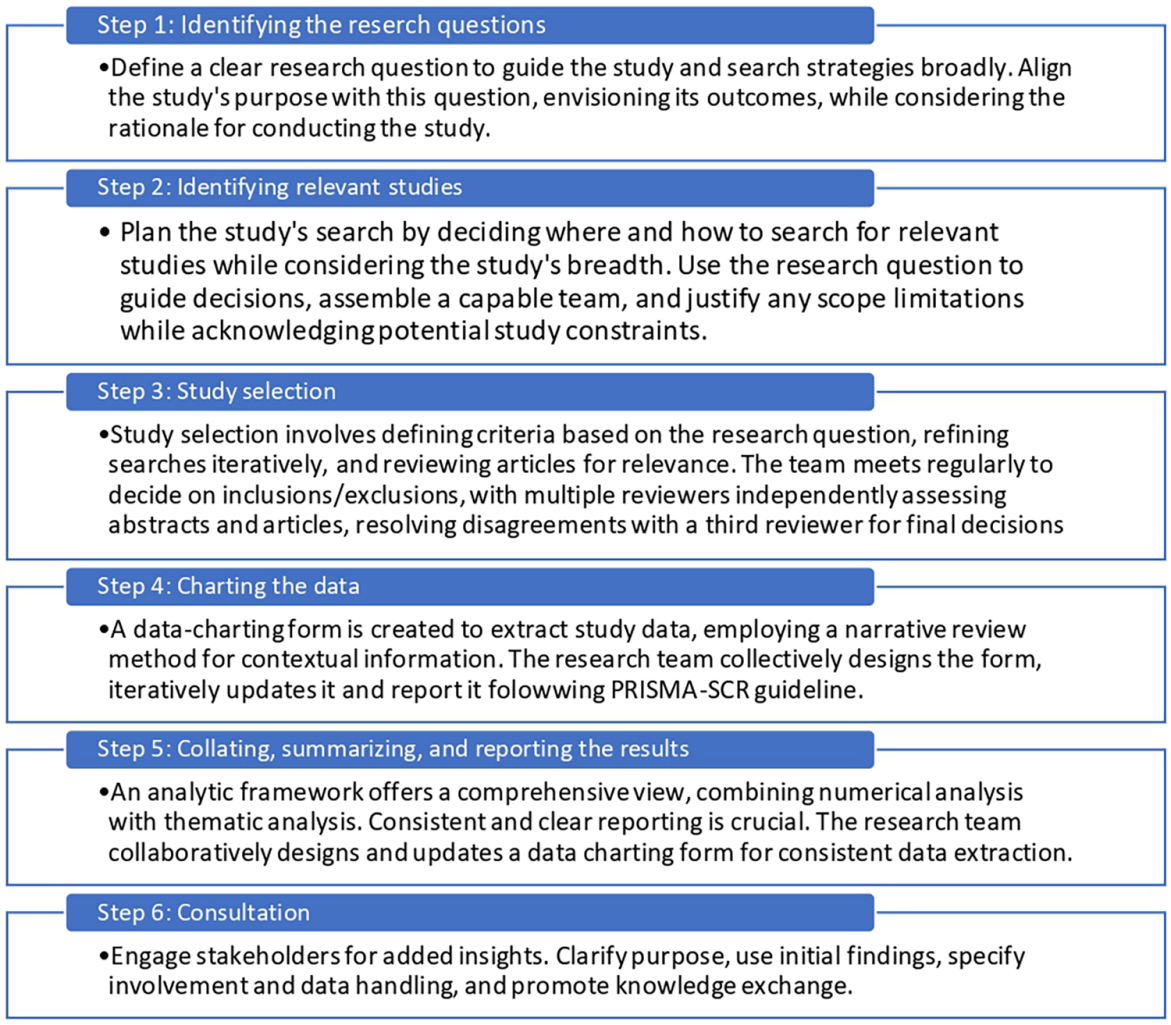
Arksey and O'Malley's (35) six stage framework for scoping reviews with Levac (36) recommendations.

This scoping review was conducted following Arksey and O'Malley (35), which was further elaborated by Levac et al. (36). We followed the guidelines on reporting by Peters et al. (37) and the scoping review extension of the Preferred Reporting Items for Systematic Reviews and Meta-Analyses (PRISMA-ScR) guidelines (38, 39) with adaptations from PRISMA 2020 (39) were followed (Figure 1). A multidisciplinary research team (e.g., rehabilitation, kinesiology, psychology) of six co-authors (researchers, graduate and undergraduate students, and one librarian) with experience in conducting literature reviews collaborated on this review.

### 2.1 Sources and search terms

The research team identified the search terms, including subject headings, that were appropriate for the different databases and keywords with truncations. It then developed a search strategy with the following concepts: (1) Individuals with mobility disability (mobility limitation), (2) Exercise (recreation or physical activity or outdoor physical activity), (3) Adaptive equipment (or sports equipment), (4) Community participation (or social participation). The specific search strategies were based on the Population, Concept, and Context framework (40). Four electronic databases, Medline (Ovid), Embase (OVID), CINAHL (EBSCO), and PsycINFO (EBSCO), were searched without any limitation on the date of publication in April 2023. These searches were regularly updated until September 2023, using the search strings detailed in Table 1. Otherresources were also searches, such as grey literature (Google Scholar and Research Gate), forward and backward citation searches, by hand searches across related journals (Journal of the Association of Computing Machinery-ACM), and conference proceedings. All data sets were uploaded and screened by two researchers using Covidence software (41).

**Table 1:** Search strings and search terms.

1 = Primary Search Term	Exercise/ or walking/ sports/ or bicycling/ or golf/ or hockey/ or mountaineering/ or snow sports/ or skiing/ or sports for persons with disabilities/ or exp water sports/ OR (exercis* or walk* or biking or cycling or golf or hockey or mountaineering or skiing or canoeing or paddling or outdoor activit* or physical activit*).mp. AND disabled persons/ or amputees/ or persons with mental disabilities/ or para-athletes/ mobility limitation/ wheelchairs/ OR [disabl* adj3 (person* or people or individual*)].mp. (para-athlethe* or amputee* or wheelchair*).mp. ((mobility or walk*) adj3 (impar* or difficult* or limit*)).mp.
2 = Secondary Search Term	Adaptive equipment.mp. OR sports equipment/ OR environment.mp.
3 = Tertiary Search Term	Community participation/ OR independent living/ or social participation/ OR [(community or consumer or public) adj3 (involvement or participation or consult* or engage*)].mp.
Final	1 AND (2 OR 3)

### 2.2 Selection criteria

The inclusion criteria for selecting resources were as follows: (1) Focused on adult outdoor physical activity or recreation provided to people with mobility disability; (2) Providing information on the perspective(s) of designers/providers/users on program accessibility/usability/inclusivity. All published empirical studies were included if they were published in English, regardless of the methodologies they applied (quantitative, qualitative, or mixed methods). Exclusion criteria were studies (1) focused on hospitals or other health facilities, (2) focused solely on clinical outcome measures, and (3) in languages other than English.

### 2.3 Selection

The selection process for this study consisted of four steps (36): (1) Identification of all relevant studies in the literature, (2) screening of all studies by applying the inclusion and exclusion criteria to titles and abstracts, (3) determining of the eligibility of the remaining studies by applying selection criteria to the complete papers, and (4) inclusion of relevant studies by re-applying the criteria to the papers in their entirety during data extraction. The Covidence software (41) automatically removed potential duplicates after all relevant databases were searched (Step 1, Identification). In the second, third, and fourth steps (screening, eligibility, and inclusion), studies were independently assessed by two researchers (first author and a research assistant) and completed the review and documented each time the reason for exclusion.

### 2.4 Charting the data

The first author identified the following variables to be extracted from the included studies: (1) the general characteristics of the study (title, author(s), year of publication, country of the study), (2) the type of outdoor activity researched, (3) the target population and the type of their disability, (4) the study design, classified as quantitative (seven designs) or qualitative (five designs) (42, 43), (5) the study aim, (6) the identified barriers and facilitators, and (7) the design recommendations.

The data from the qualitative studies were extracted by reading and re-reading their content, by extracting quotes from the individual studies, and by noting themes and categories. The data from the quantitative studies were extracted into an Excel sheet and then analyzed descriptively. A thematic synthesis approach was used by the first author to code the data. For qualitative studies, the key themes identified in the results section of each article were entered into an Excel database and then manually coded to reflect broader categories. Afterward, these categories were classified according to their conformity with the SEM. The mentioned process conducted by one person (first author). To validate the charting of the data, all of these extracted data were discussed in meetings with co-authors (WCM and WBM).

#### 2.4.1 Charting of factors within the SEM and design principles within UD

The extracted barriers and facilitators, informed by the SEM, are categorized into the following levels: (1) Intrapersonal level, the characteristics of an individual; (2) social environment, including (2a) interpersonal factors; and (2b) institutional or community factors, (3) physical environment, including (3a) natural environment factors, (3b) built environment factors, (3c) adaptive equipment, and (4) policy-regulatory factors. Often, the facilitators were simply the opposite of the barriers. The term “factors” has been utilized to encompass all these conceptualizations to streamline this study's results. The extracted design suggestions are categorized based on their coverage of the seven principles of UD, including: (1) equitable, (2) flexible, (3) simple and intuitive, (4) perceptible information, (5) tolerance for error, (6) low physical effort, and (7) size and space for approach and use. The factors were extracted by one person, first author, and then validated during meeting with co-authors.

## 3 Results

**Figure 2:**
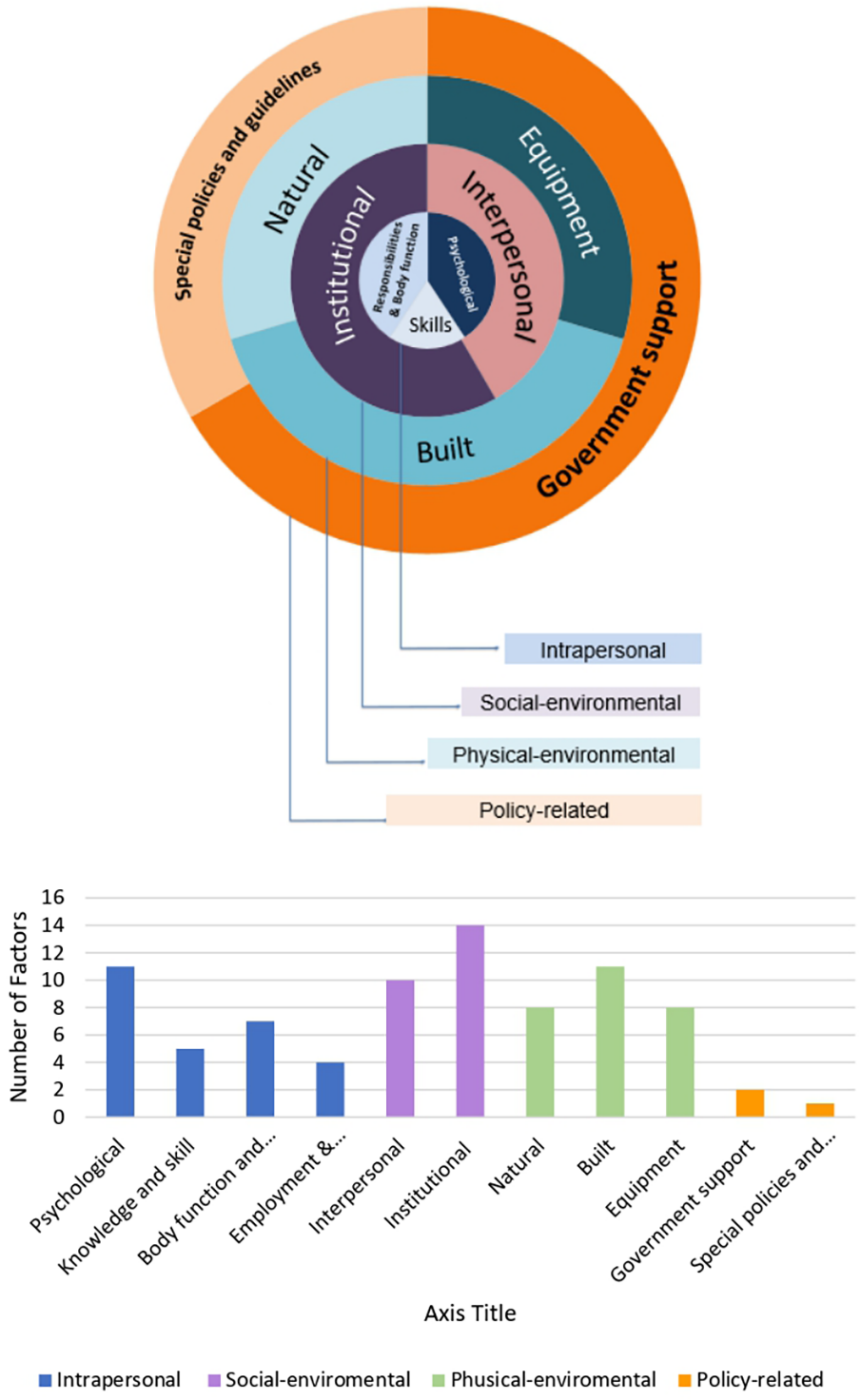
PRISMA flow chart.

After applying the inclusion and exclusion criteria, 20 studies (0.45%, *n* = 20/4,449) were included in the study. The PRISMA flow chart is presented in Figure 2. The included studies and their details are shown in Table 2. Most studies were conducted in the US (42.1%; *n* = 8) and Canada (31.6%; *n* = 6), whereas the remaining studies were conducted in Europe (21.1%; *n* = 4 in the UK, Germany, and Denmark) or Australia (5.3%; *n* = 1). Ten (52.6%) of the studies were published in or after 2019. Seventeen studies were published in peer-reviewed journals, and 2 studies were dissertations.

**Table 2:** Characteristics of included studies.

References	Year of publication	Type of study	Participants	Number	Type of activity/equipment	Aim(s)
Lawrason et al. (44) CA &USA	2022	Quantitative	Spinal cord injury ambulator	43	Community outdoors and/or indoors	Identify behavior change factors related to LTPA in SCI ambulators.
Merrick et al. (45) CA	2021	Qualitative	Children and adults requiring physical, cognitive, and/or psychosocial supports	26	Adapted paddling programs	Explore the experiences of participants
Alsaleem et al. (46) USA	2020	Qualitative	Individuals with spinal cord injury- Tetraplegic	9	Tetra-Sail	Describe the iterative design process of designing an adaptive sailing program.
Alsaleem et al. (17) USA	2020	Qualitative	Individuals with spinal cord injury- Tetraplegic	17	Tetra-Ski & Tetra-Sail	Explore the experience of individuals using shared-control and ability-based design principles.
Menzies et al. (47) CA	2020	Qualitative- Semi-structured interviews	Participants who need to use a manual wheelchair for at least 4 h a day	15	Various outdoor recreation	Explore experiences and identify the perceived barriers and facilitators
Corazon et al. DK (48)	2019	Qualitative	Individuals with mobility disability	24	Various outdoor recreation (green spaces)	Examine experiences and related constraints
Alsaleem et al. (49) USA	2019	Qualitative	Individuals with spinal cord injury- Tetraplegic	8	Tetra-Ski	Describe the iterative design and field evaluation of Tetra-Ski.
Everett (50) USA	2019	Mixed methods study	Individuals with disability	1,746	Non-motorized boating	Explore the barriers to participation
Mavritsakis et al. (51) CA	2019	Qualitative	Individuals with disabilities- Past and current users of adaptive snow equipment	20	Adaptive snow sports	Explore the experiences and factors that impact participation
Labbe et al. (52) CA	2019	Qualitative	Individuals with disabilities, staff, volunteers	36	Various outdoor recreation	Evaluate the benefits and explore facilitators and barriers to participation
Labbe et al. (6) CA	2019	Qualitative	Sailors with disabilities, staff, and volunteers	38	Adaptive sailing program	Explore the experiences, identify the perceived benefits of participating, and explore facilitators and barriers to participation
James et al. (53) CA	2018	Qualitative	Individuals with disabilities, staff, and volunteers	20	Adaptive hiking program/ TrailRider	Explore the experiences of users and nonusers of the program
Darcy et al. (54) ASTL	2017	Quantitative	Individuals with disabilities	1,046	Various outdoor recreation	Examine the barriers to sport participation
Burns et al. (14) UK	2013	Qualitative	Individuals with disabilities and support assistants	56	Various outdoor recreation	Explore participants’ views and experiences of outdoor recreation
Burns et al. (55) UK	2009	Qualitative	Individuals with disabilities	56	Various outdoor recreation (woodland and countryside leisure visits)	Explore participants’ experience
Freudenberg et al. (56) GER	2009	Quantitative	Anglers with physical disabilities	775	Recreational fishing	Identify and compare benefits and barriers to participation experienced by anglers with and without disabilities
Goodwin et al. (57) USA	2009	Qualitative	Adults with spinal cord injury	4	Hiking (TrailRider)	Understand the experience of participating
Burns et al. (58) USA	2007	Quantitative	Individuals with disabilities and their household	336	Various outdoor recreation (national forest visits	Examine the perceived barriers of participants in relation to the presence of a person with a disability in one’s household.
Williams et al. (13) USA	2004	Quantitative	Individuals with and without mobility disabilities	585	Various outdoor recreation	Describe and compare the outdoor recreation participation patterns of individuals with mobility disabilities with those of individuals without disabilities, and report on the differences between these two groups in terms of constraints to participation
Gransee (59) USA	2002	Qualitative	Women with physical disabilities	19	Various outdoor recreation	Explore constraints and strategies for the participation of women with physical disabilities in outdoor recreation

### 3.1 Key factors related to physical activity

**Figure 3:**
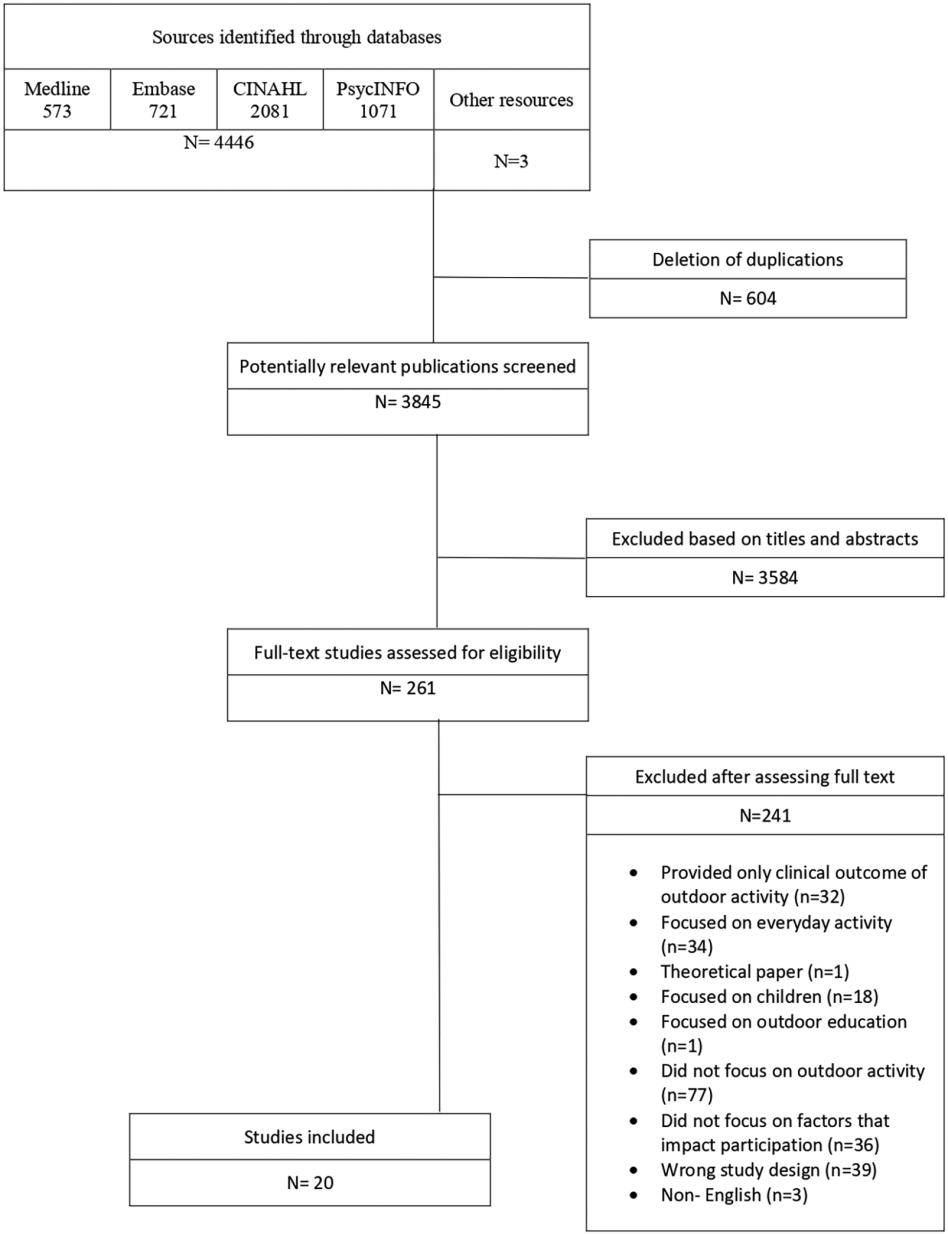
(**A**) The socio-ecological model. (**B**) Distribution of factors by category.

Overall, 37 factors were extracted from the included studies and categorized into 4 levels and 11 sub-levels. The frequency of factors in each category is shown in Figure 3B, and the details of each of its levels and sub-levels are described below.

#### 3.1.1 Intrapersonal level

The factors at this level were categorized into four sub-themes (Table 3): psychological, knowledge and skill, body function and structure, and employment and responsibilities, i.e., family and work responsibilities. The factors most frequently mentioned by studies were *psychological factors* describing the emotions and personality types of individuals, self-perceptions, attitudes, and perceived benefits of outdoor activity. Mainly, character traits of individuals with challenge-seeking personalities, positive attitudes toward outdoor physical activity (e.g., finding friends, enjoying being in nature, and wanting to improve function), and toward themselves (e.g., self-confidence) were identified as facilitators. On the other hand, individuals with risk-averse personalities, negative attitudes toward outdoor physical activity (e.g., anxiety about socializing), fear of being embarrassed, and lack of confidence were more likely to experience barriers to participation in outdoor adapted activities. One study (45), which included recreational and therapeutic programs, indicated that if the participants' goal of joining the outdoor activity matches the program goals, it could act as a facilitator for participation. Nevertheless, it further underlined that enjoying the activity was an essential facilitator for all participants.

**Table 3:** Intrapersonal factors.

Psychological	Personality (risk aversion vs. challenge seeking)
	Attitudes toward outdoor physical activity
	Self-perceptions
	Congruence between activity/program and personal goals
Knowledge and Skill	Assistive device skills
	Familiarity with outdoor activities
	Awareness of the availability of adaptive programs
Body Function and Structure	Physical capabilities
	Health outcomes of activities
Employment & Responsibilities	Financial capabilities
	Responsibilities and time constraints

In the *body function and structure* subtheme, physical limitations of individuals, such as types and levels of impairment, were mentioned as factors that influence participation. Participants in the included studies stated that as a result of their impairment, they experience fatigue, an inability to know how they would feel on a particular day, and an inability to regulate their body temperature in outdoor spaces properly. Moreover, some individuals mentioned physical health consequences of participation in outdoor activities, such as back pain due to rough and bumpy pathways, as a barrier to their participation. In contrast to this, having knowledge and skills in the handling of assistive devices, such as high wheelchair skills and a familiarity with outdoor sports, helped some individuals enjoy their participation more.

One of the themes that emerged from the included studies was the impact of work and the financial situation of individuals with mobility disabilities on their participation in outdoor adaptive activities. The last subtheme deals with the *employment status and responsibilities* of individuals with mobility disabilities. On the one hand, an individual with a mobility disability who works or has a responsibility to their family mentioned lack of time as a barrier to their participation. On the other hand, low no income and financial instability in general are barriers to participation in an outdoor adaptive activity. These findings suggest that individuals with mobility disabilities have different needs and preferences when it comes to engaging in outdoor adaptive activities and that these factors should be taken into account when designing and implementing such programs.

#### 3.1.2 Social environment level

##### 3.1.2.1 Interpersonal

On the interpersonal sub-level (Table 4), factors were categorized into two sub-themes: social support and attitude. *Social support* can come from families, friends, peers, and other participants of the program. On the one hand, this support can be a form of encouragement to participate, a companionship, and assist them during the program or sports activity, for example, if it is a joint family activity such as skiing. On the other hand, a negative *social attitude*, such as low expectations of the abilities of people with disabilities and stigmatization by onlookers during the program, could be a barrier for individuals with mobility disability.

**Table 4:** Social Environment factors.

Interpersonal	Social support	Having companions (family, friends, others)
		Social circle’s support of outdoor activity
	Social attitudes	Stigma (e.g., being stared at)
		Expectations of the abilities of individuals with disabilities
Institutional	Staff and volunteers	Knowledge and competency of staff and volunteers
		Supportive attitude of staff and volunteers
		Number of available staff and volunteers
		Dissenting opinions of organisers on health and safety aspects
	Program delivery and logistics	Complexity of booking and scheduling
		Organization of daily operation
		Amount of planning and time an activity takes
		Amount of equipment per program
		Cost of program
		Amount of information or insight during designing
		Assessment of PWD’s needs
		Extent of the independence provided
		Customization options of an activity to fit the members’ needs
		Focus on the members’ strengths
		Amount of safety precautions implemented by organizations

##### 3.1.2.2 Institutional and community

In this level (Table 4), the factors most frequently mentioned as facilitators in studies were availability, knowledge, competency, and attitude of the volunteers and staff. Another factor that positively affects participation is assessing the needs of individuals with disabilities and gathering sufficient information or insight during program design and implementation. For example, users were eager to participate in customized activities that fit their needs and focused on their strengths. In some programs, they were not satisfied with the activity because they felt it did not meet their desired level of independence (14, 45, 47, 52, 53). An additional factor mentioned in two studies (6, 52) was the importance of a simple and organized booking process and scheduling of the program's procedure.

#### 3.1.3 Physical environmental level

This level consists of factors categorized in the following sub-themes: physical (natural and built) environment and adaptive equipment (Table 5).

**Table 5:** Physical Environment factors.

Natural	Effect of green and blue space
	Weather condition
	Terrain condition
Built	Safety of the environment (e.g., the facilities or paths are shared between program participants and able-bodied individuals)
	Access to necessary infrastructures (e.g., washroom, elevator, etc.)
	Access to the location (e.g., long distance, direct public transportation, pay for parking)
Equipment	Handling of equipment (weight and size)
	Availability of specialized leisure equipment
	Diversity of equipment
	Cost of equipment
	Flexibility of equipment (e.g., seasonal usage)
	Maintenance requirements
	Storage requirements
	Access to local equipment
	Accommodation of various ergonomic needs
	Need for assistance during installation and use

The *physical environmental* factors that affect the participation of individuals with mobility disability in adaptive outdoor physical activity are part of the *natural* or the *built* environment. The positive effect of blue and green space on physical and mental health is mentioned as a great facilitator for participants returning to a program (45, 47, 51, 58). However, weather conditions (e.g., rain, snow, or cold temperatures) and terrain conditions (such as poor trail conditions and a general steepness) result in a situational impairment that limits the use of adapted programs and presents barriers to their design and development.

Hosting adaptive physical activities in nature usually leads to a great distance from the homes or workplaces of the participants, and the lack of accessible public transportation to the facilities was frequently cited as a barrier in studies. One study (53) suggested using a transportation system exclusive to individuals with disabilities; however, unreliable arrivals and departures and the possibility that the transportation schedule may not coincide with the time of the outdoor activity is still a barrier to individual participation. Moreover, three studies (6, 14, 60) mentioned a lack of necessary infrastructure at the location of the outdoor activity, such as accessible washrooms or elevators. In one program (14), sharing facilities and pathways with abled-bodied users negatively affected the implementation of the adaptive program and, in some cases, was perceived as risky.

The *adaptive equipment* used in a program was mentioned as a factor in 8 studies (6, 45, 47, 48, 54, 56, 60). Although the present study does not aim to focus on the details of the design of the equipment, the following factors are extracted from included studies. Participants found a program's high diversity and specialized equipment to be a facilitating factor. One study (6) mentioned the importance of regular maintenance of the equipment. In addition, four (6, 45, 48, 60) studies were concerned with the logistics of the equipment, such as the limitations of getting in and out of with a low seat rear-wheel hand cycle; another one mentioned the significance of the equipment's ergonomics, specifically the posture of the user when using the equipment. On two occasions (45, 54), participants wanted to purchase their own equipment, but its high price and the required storage space needed for the equipment, which can only be used during a specific season, was the barrier that prevented them from doing so.

#### 3.1.4 Policies and regulations level

The factors categorized at this level (Table 6) directly relate to policies at the governmental level. Two main factors emerged from the included studies that fall directly under the purview of government policymakers, including government support (mostly financial support) and health and safety regulations and guidelines. Participants of two studies (14, 45) mentioned the importance of safety regulations. When the regulations are restrictive (such as asking for special insurance), it affects the autonomy and perceived risk of people with disabilities in outdoor activities.

**Table 6:** Policies and regulations factors.

Government support (e.g., financial support)
Special policies and guidelines (e.g., restrictive policies in recreation, demand for special insurance)

### 3.2 Designing for adaptive outdoor physical activity and UD principles

At this point in this research, both designs implemented by studies and designs recommended by users during qualitative interviews to maximize the inclusion and accessibility of adaptive outdoor physical activity were identified. The seven principles of UD were used as an analysis tool to map the recommendations.

Nine studies identified design recommendations on the accessibility aspect of adaptive outdoor physical activities. These recommendations are closely aligned with some of the seven UD principles for adaptive outdoor activity design. Autonomy and independence as overarching principles that characterize UD were specifically mentioned in one of the studies (45). Participants suggested adding additional active elements to the equipment to make it more independent. In the following section, the most prevalent suggestions from the 9 studies, grouped into seven principles, will be named and expanded upon.

*Principle 1-Equitable Use*: Three of the proposed recommendations were on the intrapersonal level, including advertising a program through the local health center to provide equitable knowledge about its existence, and lowering the cost of the program to provide equitable access to low-income individuals. One study (45) suggested designing equipment with the same appearance as equipment of able-bodied users. This design aims to mitigate the interpersonal barriers and stigmatization encountered by users of adaptive equipment during interactions with observers and onlookers. Another recommendation (47) was to rent shared equipment to simultaneously address the barriers of the high cost of adaptive equipment, the need for storage, and its seasonal usage. This would also help ensure that users are not constrained by program schedules and restrictive policies and improve their independence (47).

*Principle 2-Flexible Use:* Two design suggestions are detailed in this section. Both aimed to improve the flexibility of the adaptive activity by making the equipment more flexible. That is, first (17), to design the equipment in a way that it can be customized and adjusted for all types of mobility disabilities, as recommended in two studies. For example, one of the studies (49) designed a sailboat called the Tetra-Sail, which is a novel sailing system that utilizes a Shared-Control paradigm to combine commands from both a primary user and a skilled adaptive trainer (control collaborator). This study mentioned that a wheelchair system can have a variety of adaptations and customizations, depending on the health condition of its user, many of which should also be included on Tetra-Sail. Their design on the sailboat featured basic elements such as seat adaptability, padded cushions to decrease pressure, straps to keep different body parts in place, holders for drinks and medication, and additional space for any health equipment a user might need (e.g., an electrical ventilator). The second suggestion (51) is to design the equipment in such a way that users have the option to choose their preferred level of autonomy, which will result in them gaining more self-confidence. An example of this suggestion is mentioned in one of the studies (53), which aims to explore the experiences of users participating in an adapted hiking program that utilizes a specialized mobility device called the TrailRider. The TrailRider is a one-wheeled chair equipped with handles and brakes, enabling individuals with disabilities to access natural environments. It is employed in adapted hiking programs worldwide, where volunteers aid riders in navigating challenging landscapes. Attaching a steering wheel to the TrailRider to turning it into an off-road wheelchair and the Shared-Control design of the Tetra-Sail, are examples of transferring control to the users. Creating multiple levels of control allowed for a variation in difficulty and made it possible to design a challenge that matches the skill level of the participants, which is an important factor in the enjoyment and performance of physical activities.

*Principle 3-Simple and Intuitive:* Two suggestions were brought forward for making an outdoor activity simple and intuitive, where volunteers played an important role. The first suggestion (45) was to use a peer mentor with a mobility disability and the experience to accompany staff or volunteers during the program. An individual with the same disability can understand the difficulties that users encounter better and can transfer knowledge and experience to users more effectively. The second suggestion (49) was related to the presence of a knowledgeable volunteer who gives the user feedback on equipment use during and after completing a task. Getting feedback and communicating with staff in general, as mentioned in other studies, will be discussed in detail in the next principle.

*Principle 4-Perceptible Information:* Effective communication between the user and a trained partner, who gives the user real-time information and assistance based on their performance, is essential. Transparent communication one of the principles of an ability-based design approach and was used in the Shared-Control Tetra-Sail program (46). In this program, a person with a tetraplegic spinal cord injury is coupled with an assistant to perform the sailing activity. The features and functions of the activity are divided between the individual with a disability (user) and the partner (control partner). For more effective communication between the user and the control partner, the designers added a Bluetooth speaker to provide feedback on the user's sip-and-puff commands. However, ensuring effective communication can be a challenge to some degree in other contexts, such as the Tetra-Ski (49). For example, in this instance, it is possible for the controlling partner to call out short phrases to the participant while skiing, but the participant has limited ability to communicate back to them. To complicate things further, while the control partner is able to see and respond to the general movements of the participants on the joystick, this is not possible when equipment such as a sip-and-puff device is required.

*Principle 5-Tolerance for Error*: Individuals with a mobility disability are often seen as being “at risk” outdoors. This can be a barrier to their participation, no matter if it is a result of their own fear of being at risk (intrapersonal barrier) or because of societal (interpersonal) and institutional perspectives (environmental barrier). Multiple design strategies were provided by the studies that are included in the present scoping review to minimize the risk of participation of individuals with a mobility disability. A study on Tetra-Ski (49) addressed this issue by sacrificing autonomy in exchange for their safety. The designers limited their customization options to a predefined set of options, as certain user preferences can lead to dangerous situations. The alternative strategy was to shift equipment control back and forth between the trainer and participant to find a safe setting with the right amount of control that worked for both of them. In one study (49), a simulation of the program was conducted prior to the actual program to assess the level of safety for each individual based on the function of each participant. If the participant demonstrated limited function and mastery of the device during the simulation session, the trainer would be in control of the equipment. Generally, having reliable volunteers, constant communication between volunteers and staff members, and a reliable assessment of weather conditions by staff were important strategies that facilitated the creation of a safe environment for activities. In another study (6), the safety of the program was ensured through the use of special equipment, such as using specific materials for a boat to prevent it from sinking, as well as maintaining regular maintenance intervals by assigning a specific employee to be in charge of repairs.

*Principle 6-Low Physical Effort:* Studies provided five design suggestions to make adaptive outdoor physical activities efficient, comfortable, and low in fatigue. All of them suggested modifications to the equipment, including (1) reworking seats to account for scoliosis, spasticity, and improve trunk control (53), (2) changing the design of hand cycles to be rear-wheeled or high-seated to facilitate mounting and dismounting (53), (3) lighter equipment to provide access to a trail in the woods and a beach (47), (4) adding a removable front wheel to facilitate movement on rough or soft terrain by raising the casters of a wheelchair (47), and (5) providing a basic and advanced option for the equipment to let the user switch between them in case of fatigue (49).

*Principle 7-Size and Space for Approach and Use:* To guarantee adequate space for individuals with mobility disability, studies suggested the installation of a floating dock to facilitate launching a boat and smaller equipment to make turning and maneuvering on trails easier (6, 53). In terms of the scope of designing equipment, it's worth mentioning that the majority of the studies primarily focused on designing equipment and interventions for specific adaptive activities. Five studies were related to adaptive sailing (6, 17, 46), paddling (45), or fishing (56), three studies focused on snow sports (51) and skiing (49, 56), and only two studies explored adaptive hiking (53, 57), both of which examined the TrailRider design. While there were studies that examined various outdoor activities, they did not provide specific design suggestions for equipment.

## 4 Discussion

This scoping review of peer-reviewed and non-peer-reviewed studies shows that the factors that impact the participation of individuals with mobility disability in adaptive outdoor physical activity can be categorized, informed by an SEM, as intrapersonal, social environmental, physical environmental, and policy-related. The results of this review suggest that each of these factors can act as a barrier or a facilitator. To improve the participation of individuals with mobility disability in adaptive outdoor physical activities, barriers that exist at multiple levels of the model must be addressed, and facilitators must be maintained or newly established. In the context of the present scoping review, design suggestions to address these barriers were also mapped based on UD. These design suggestions are discussed below, along with the most prominent factors identified by this review and the relevant interventions suited to increase outdoor physical activity participation of individuals with a mobility disability.

### 4.1 Intrapersonal factors

Intrapersonal factors were the most frequently identified factor that influenced the participation of individuals with a mobility disability. These factors included a large range of aspects, such as attitude, emotion, behavior, and self-perception. Design suggestions aimed to make programs more flexible to (1) give users with a mobility disability the option for more autonomy, which leads to more self-confidence, and (2) provide tailored equipment and a safe environment, which helps to reduce the user's perceived risk of an outdoor activity. However, none of the included studies mentioned applying any behavioral theories to facilitate participation in adapted outdoor physical activity, despite there being evidence in favor of it at the psychological level within behavior change theories and models. For example, the HAPA (52) contains constructs that include concerns about potential behavioral risks (i.e., risk perceptions), self-perceptions (i.e., self-efficacy), attitudes toward outdoor physical activity (i.e., outcome expectations), and strategies for behavior change (i.e., action planning). This study encourages combining SEM with different theories of behavior change to explore how psychological factors affect the participation of individuals with a mobility disability in adapted outdoor physical activity and how different designs can facilitate participation.

Factors mentioned in the chapter about *Body Function and Structure* were the second most frequent factors. These factors encompass experiencing fatigue due to their impairments and the physical health consequences of outdoor activities resulting from non-inclusive environments and equipment. By connection the identified limitation with UD principles, Design suggestions with the aim of ensuring a low physical effort (principle 6 of UD) sought to reduce the level of fatigue and secondary health concerns resulting from adaptive outdoor activities. Previous studies on the physical activity of individuals with mobility disabilities mentioned that body function and structure factors could completely prevent participation or affect the types of activities a user can take part in (16). This review agrees with those findings and suggests that the extent of body function also has the potential to limit the user's autonomy. For example, in a shared-control ski program (49) for individuals with tetraplegia, the trainer would take over complete control if the medical staff suggested that an individual is unable to safely control the ski on their own. From this, it follows that the level of the user's body function decides how much control they have over their ski, thus affecting their independence.

To better understand how individuals' independence is impacted, it is essential to examine how studies and participants with mobility impairments define independence. In the majority of studies, dependence is often defined in line with the common-sense understanding of being unable to perform tasks oneself, leading to reliance on others to accomplish some or all desired activities (61), such as transferring or using assistive devices (14, 45, 47, 52, 53). Conversely, independence implies self-reliance, where individuals do not require assistance from others. Despite the absence of explicit measurements for independence in these studies, Burns et al. (14) referenced Oliver's concept of enforced dependency (1993) (61), where he argued that societal and economic structures, rather than impairments themselves, render individuals with mobility impairment dependent on others. Furthermore, he commented on the definition of independence as a complete self-reliance that within a modern industrial society, absolute independence is a concept that doesn't apply to anyone, given our mutual interdependence (61). Hence, the dependence of people with mobility impairment, is not a unique feature which sets them apart as categorically from the rest of the population but rather distinguishes them by different levels of dependency.

When considering the level of dependence, it is important to differentiate between independence and interdependence. The former enables individuals with disabilities to make their own decisions through support systems. However, some researchers challenge the notion of independence as the ultimate accessibility goal. They argue that everyone relies on others to some extent and emphasize that self-sufficiency can harm people, particularly those with disabilities. Instead, the researchers advocate for interdependence to achieve access. For example, one can argue that the shared-ski program is an application of the interdependence frame due to the relationship between user and trainer, adaptive device, and environment to create accessibility and improve quality of life. Independence and interdependence should not be viewed as opposing or incompatible concepts; instead, they complement each other.

Limited time and income were the third most frequently mentioned intrapersonal factors in the reviewed studies that pose barriers to participation (45, 52). This barrier is already identified by many previous studies on the physical activity of individuals with a mobility impairment (16, 47, 52, 62). Designs that attempted to make a program more equitable, for example, by renting shared devices and offering the program at a low price, sought to address the barrier of low income. It is important to emphasize the importance of equitable cost of programs because financial barriers were found on two levels: first, the barrier of low income on the intrapersonal level and, second, the cost of the equipment and the program itself on the environmental level.

### 4.2 Social environment level

#### 4.2.1 Interpersonal factors

This study identified social support and social attitude factors on the interpersonal level. Although most of the studies mentioned these barriers, only one of them (45) made a suggestion on how to reduce the stigma toward outdoor activity of individuals with a mobility disability by introducing the social-relational model to the discussion. In this model, disability is conceptualized as a result of relationships with other people and structures. This model refers to the negative impact of society on an individual's participation as “social disablism,” which could affect individuals on an intrapersonal level through negative attitudes, unsupportive behavior, and insensitive comments. Social disablism can damage self-perception and limit what individuals with disabilities think they can achieve (e.g., participating in sports) and, subsequently, what they are convinced they can become (63). In light of the substantial consequences of social disablism and the identified lack of design suggestions to address this issue, this review recommends researchers, designers, and policymakers prioritize the mitigation of social disablism by considering the identified intrapersonal factors in their work. Examples of this would be to make adaptive device more visible in society by having them offered by more institutions and facilities, thus decreasing the stigma connected to them, and helping individuals with disabilities to improve and increase their social circle.

#### 4.2.2 Institutional factors

The factors mentioned most frequently in the social, environmental, and institutional subtheme were the knowledge and competency of staff and volunteers as well as the insight and knowledge of program designers about user needs, which have often been lacking to date (63). Our study found a gap in design suggestions in the included studies to improve staff's knowledge and competency. This is in line with other studies (16) that stated that recreation staff and volunteers are frequently criticized for their lack of skills and knowledge in adaptive activities, inclusive environment creation, and guidance or exercise instructions (64, 65). In addition, the knowledge and competency of staff and volunteers could impact other accessibility factors, such as intrapersonal ones (attitude toward outdoor activity and self-perceptions). The lack of attention to this requires policies and educational programs to improve the skills and knowledge of staff and volunteers. For example, organizing regular workshops on disability awareness, communication skills, safety procedures, and adaptive equipment for them will be helpful in tackling the issue (16). Another way of addressing these institutional barriers is by hiring a Certified Therapeutic Recreation Specialist (CTRS) (50). Recreational therapists with the certificate CTRS, certified by the National Council for Therapeutic Recreation Certification (NCTRC), have the required skills, knowledge, and abilities that are essential in recreational therapy. They are capable of implementing accessible programs, satisfying not only the participants but also the organization by demonstrating how important equal access is. Studies showed that larger organizations with greater financial resources and more CTRS among their staff had a better chance of having up-to-date policies, conducting inclusivity training and workshops with their employees, and purchasing adaptive equipment (66).

### 4.3 Physical environmental level

Our review identified that both the natural and the built environment have the potential to lead to challenges for participants of outdoor recreation with varying abilities (62). This is in line with another study that stated that park visitors and participants are more likely to return for another visit if the facilities, the infrastructure, and the parks in general are well maintained (67). One of the studies (68) mentioned a design suggestion that would allow users with a wheelchair to cross soft ground and sandy beaches; however, this does not relieve the management of parks and public areas of their responsibility to ensure accessibility. They nevertheless play a crucial role in the ability (or lack thereof) of individuals with disabilities to participate in their chosen outdoor recreation activities. Thus, there is a need for standards for an inclusive outdoor environment. Recently, a scoping review showed that there is only a limited number of studies on accessibility standards for nature spaces (69). The results of the scoping review (70) and multiphase study protocols (71) on barriers and facilitators, as well as existing standards of the accessibility of parks that were published, could be helpful for the development of guidelines and best practices to ensure accessibility of nature and the built environment.

### 4.4 Policy level

The cost of programs and equipment was often cited as a barrier in the included studies, a challenge exacerbated by the fact that individuals with disabilities are typically at the lower end of the socioeconomic spectrum. Governmental support could address this multi-level barrier. It is worth noting that although only two factors are directly related to the policy level, many other factors can also be addressed by acting at this level, by, for example, shaping public policy to improve accessible transportation, volunteer, and staff training, and building an accessible outdoor environment (16). Considering the trend of volunteerism in PA and the need for more training for both volunteers and staff in adaptive physical activity, this study suggests that the government support this process financially and logistically. Furthermore, many volunteers have other obligations, such as full-time jobs or studies, which may limit the time and energy they have at their disposal that is necessary for adaptive PA programs and which leads to barriers to participation of individuals with mobility disability. Also, adaptive outdoor PA might require more support than other PA programs, thus leading them to rely more on volunteers in addition to paid staff. The paid staff in adaptive organizations could be responsible for administrational purposes such as recruiting, training, scheduling, and matching volunteers with individuals with mobility disability based on their skill set. There could be policies to support adaptive PA program to carry out their business in a safe and meaningful way and implementing these policies would have financial implications.

### 4.5 Mapping designs suggestions using UD

The seven principles of UD, when utilized as an analytical tool with the aim of map design recommendations, can support the design of adaptive outdoor PA. Using UD as an assessment tool should not be limited solely to the end of the design process. Such an approach, which has a tangible impact on users' well-being, may result in additional costs and time for adjustments to accommodate various situations (72). It is essential to assess usability and inclusion throughout the entire project life cycle—before, during, and after construction—by establishing an evaluation framework based on UD principles. For instance, Wu et al. (73) conducted a survey on UD for fitness wearable devices, and reviewed how and when different research efforts examined each principle to develop such devices. The researchers also elucidated how each principle could be incorporated during the design phase of wearable device creation, demonstrating the utility of user-centered approaches in the process (73). This involves alternating iterations and evaluations through methods such as focus groups, interviews, and surveys. Some studies have developed checklists to assess programs. Checklists form the core of the most formal evaluations and serve as the foundation for numerous published studies. Evaluations driven by checklists in UD rely on a set of simplified criteria, typically derived from the seven UD principles. For instance, Kim et al. (74) devised a 27-item measure based on UD principles, assessing user perspectives in sport facilities.

To the best of our knowledge, there isn't an existing evaluation framework based on Universal Design (UD) specifically for assessing outdoor physical activity programs. However, Mosca et al. (72) described their methodology on developing an evaluation framework for a building throughout various stages of an architectural project, utilizing a multicriteria decision analysis approach. This method was derived from a literature review and workshops involving stakeholders and experts (72). Based on these insights, it's suggested to create an evaluation framework based on UD principles for assessing outdoor physical activity programs. This could involve conducting focus groups and workshops with stakeholders and experts to refine and develop the framework.

Despite that, it is important to note that there are inherent limitations in labeling recommendations, especially those extracted from studies not designed with the conceptual framework of UD. Furthermore, UD strongly emphasizes the promotion and enabling of independence, individualism, and self-reliance, particularly for older individuals or individuals with disabilities (75). Some of the studies indicated that it may not be necessary to design for independence, individuality, or self-reliance. Instead, having a family member, friend, or companion share responsibility during an activity may lead to interconnection and interdependence, which may benefit both parties. This may precipitate a response shift in which people redefine what independence means to them. Nevertheless, presently, UD is still not commonly applied, for which reason there is a lack of innovation, future iterations, and evolutions of UD in relation to adaptive outdoor physical activity. Altogether, the findings from the present scoping review indicate that UD has strong potential; however, additional research is necessary to articulate how UD can ensure inclusion in adaptive outdoor recreation.

To get a better insight into the users’ needs, researchers suggested using a Collaborative-participatory design. In this iterative process, the user is positioned at the inside of the design process and acts as an active contributor during each step of the development (76). There are few studies that used this design strategy (77, 78). For example, Slingerland et al. (78) applied participatory action research while designing the Canadian Centre for Mental Health and Sport. They concluded that the design approach gave stakeholders a sense of agency and empowerment in the design and outcome of the project. Thus, this review suggests that future studies use design methods that involve users in the design process and allow them to be active contributors, and then evaluate their effectiveness in the context of adaptive outdoor design.

In the process of designing an adaptive physical activity programs, it's vital to acknowledge that barriers exist across different levels of SEM, with a majority at the institutional level. Future studies should aim to develop programs addressing barriers at various levels (e.g., intrapersonal, environmental, and etc.) within the SEM.

For instance, a program offering rental adaptive equipment can address certain intrapersonal barriers, such as financial limitations and attitudes toward outdoor activities, by providing individuals the opportunity to engage in adaptive outdoor activities with no storage requirements. Additionally, this initiative can address social environment factors by reducing stigma, cutting program costs, and offering greater autonomy in activity selection. Implementing such a design requires training of staff and volunteers for the program and infrastructure development.

For a successful program, it's crucial to consider not only environmental criteria but also institutional aspects, including staff and volunteers. The involvement of professionals, such as physical and occupational therapists, alongside experienced volunteers remain essential. To cultivate a more knowledgeable professional base, integrating relevant coursework or elective programs into their curriculum or providing opportunities for involvement in adapted outdoor programs as staff or volunteers are effective strategies to meet this need.

Additionally, this review underscores the lack of awareness of adaptive equipment, programs, and environments to enable adaptive outdoor PA and shifts attitudes away from impossibility to possibility. In fact, several studies report that rehabilitation centers expose inpatients to the possibilities that influence their awareness (79, 80). When individuals become aware of these opportunities, the likelihood of continued engagement post-discharge may be enhanced.

### 4.6 Limitation

While this scoping review was conducted vigorously and systematically, there are several limitations to acknowledge. Firstly, the literature included was limited to material published in English; for that reason, findings published in other languages have potentially been overlooked. Secondly, although this scoping review did not restrict the country of the published studies, results originated only from developed countries where urban and rural accessibility infrastructure for individuals with a mobility disability is better developed than in developing countries. Therefore, this review might underrepresent the barriers to participation in adaptive outdoor activity in developing countries. Lastly, the low number of included studies is a recognized limitation.

## 5 Conclusion

This research categorized factors that impact the participation of individuals with mobility disability, informed by SEM, as interpersonal, social, environmental, physical environmental, and policy-related, and then mapped design suggestions based on the seven principles of UD. This study showed that there are gaps in knowledge about these factors and in the designs addressing them. This study suggests conducting further studies to focus on the strategies addressing the mentioned gaps (such as at the social-environmental level) and to preferably address barriers that exist at multiple levels (such as studies about rental programs of adaptive equipment). At each level, knowledge about the most frequent barriers will be helpful for prioritizing strategies that are best suited for a new design. Finally, This study recommends that designers involve individuals with mobility disability in their design process to gain better insight into their needs.
